# Magnetic heating of nanoparticles as a scalable cryopreservation technology for human induced pluripotent stem cells

**DOI:** 10.1038/s41598-020-70707-6

**Published:** 2020-08-12

**Authors:** Akira Ito, Kantaro Yoshioka, Shinya Masumoto, Keiichiro Sato, Yuki Hatae, Tomoki Nakai, Takashi Yamazaki, Masazumi Takahashi, Shota Tanoue, Masanobu Horie

**Affiliations:** 1 Department of Chemical Systems Engineering, School of Engineering, Nagoya University, Furo-cho, Chikusa-ku, Nagoya, 464-8603 Japan; 2 Precursory Research for Embryonic Science and Technology (PRESTO), Japan Science and Technology Agency (JST), 4-1-8 Honcho, Kawacughi, Saitama 332-0012 Japan; 3 Department of Chemical Engineering, Faculty of Engineering, Kyushu University, 744 Motooka, Nishi-ku, Fukuoka, 819-0395 Japan; 4 Technical Department, Dai-Ichi High Frequency Co., Ltd., 1-45 Mizue-cho, Kawasaki-ku, Kawasaki, 210-0866 Japan; 5 Division of Biochemical Engineering, Radioisotope Research Center, Kyoto University, Yoshida Konoe-cho, Sakyo-ku, Kyoto, 606-8501 Japan

**Keywords:** Stem-cell biotechnology, Biomaterials - cells, Nanoparticles

## Abstract

Scale-up of production is needed for industrial applications and clinical translation of human induced pluripotent stem cells (hiPSCs). However, in cryopreservation of hiPSCs, successful rewarming of vitrified cells can only be achieved by convective warming of small volumes (generally 0.2 mL). Here, we present a scalable nano-warming technology for hiPSC cryopreservation employing inductive heating of magnetic nanoparticles under an alternating magnetic field. The conventional method by water bath heating at 37 °C resulted in a decrease of cell viability owing to devitrification caused by slow warming of samples with large volumes (≥ 20 mL). Nano-warming showed uniform and rapid rewarming of vitrified samples and improved viability of hiPSCs in the 20-mL system. In addition to single cells, hiPSC aggregates prepared using a bioreactor-based approach were successfully cryopreserved by the nano-warming technique. These results demonstrate that nano-warming is a promising methodology for cryopreservation in mass production of hiPSCs.

## Introduction

Pluripotent stem cells, including human embryonic stem cells (hESCs) and induced pluripotent stem cells (hiPSCs), are a promising cell source for regenerative medicine because of their unlimited proliferation potential and differentiation capability^1,2^. The first patient was treated with an hESC‐based cellular therapy product in a clinical trial for spinal cord injury in 2010^3^. In 2014, the first patient received hiPSC-derived retinal pigment epithelial cells for macular degeneration^4^. Although pluripotent stem cells have been generated at laboratory scale, large scale production processes by standardized and economically viable procedures and technologies will be required^5^. One of the challenges in large scale expansion of pluripotent stem cells is suspension culture of cell aggregates in stirred bioreactors^6,7^ in which single cells form cell aggregates in the presence of the small molecule Y27632 (Rho-associated coiled-coil kinase inhibitor)^8^. Furthermore, bioreactor-based suspension culture of pluripotent stem cells can be used for large scale induction of functional cells such as iPSC-derived cardiomyocytes^9^. In addition, a recent report suggests that cell aggregates can be used as building blocks for tissue engineering^10^.

One bottleneck in manufacturing pluripotent stem cells is robust cryopreservation, and large-scale cell cryopreservation will be mandatory for industrial applications and clinical translation. Cryopreservation is classified into two distinct methods: slow freezing and vitrification. In vitrification methods, cryoprotectant solutions with high cryoprotectant concentrations are used, and cells are rapidly frozen by direct immersion of the container in liquid nitrogen. Originally, vitrification methods were developed for cryopreservation of oocytes and embryos^11^. Fujioka et al*.* developed DAP213, a cryoprotectant solution containing dimethyl sulfoxide (DMSO) for primate ESCs, and succeeded in cryopreservation of ESCs by vitrification of a 0.2-mL scale^12^. However, Katkov et al*.* reported that cryopreservation with DMSO diminished expression of the pluripotency marker Oct3/4 in ESCs^13^. More recently, Ota et al*.* developed a DMSO-free cryoprotectant solution called StemCell Keep^14^ in which carboxylated ε-poly-l-lysine was added as a cryoprotectant. StemCell Keep showed successful cryopreservation of mesenchymal stem cells^15^, hESCs^14^ and hiPSCs^16^. However, the size limitation remains and the 0.2-mL scale (1 × 10^6^ cells/mL) is the gold standard for vitrification of hiPSCs.

In the cryopreservation process, both cooling and warming rates should exceed the critical cooling rate (CCR)^17^ and critical warming rate (CWR)^18^ to avoid failure of vitrification, resulting in cell damage caused by ice crystal growth^19^. In large scale cryopreservation, one of the major technological barriers is achieving the CWR to avoid devitrification, because the conventional method of rewarming is performed by immersing vitrified cells in a water bath at 37 °C to avoid cellular damage caused by overheating. This leads to a low warming rate at the centre of large volume samples with large diameters. Additionally, high temperature gradients between the centre and edge of large volume samples may cause cracking and devitrification. Therefore, technological breakthroughs to overcome slow and uneven rewarming are required for large-scale cryopreservation of human iPSCs.

Because magnetic nanoparticles have unique features including magnetic attraction, they have been used for medical applications^20,21^, including the processes of regenerative medicine, such as magnetic cell separation, gene transfection, cell patterning, and tissue engineering. Another unique feature is that magnetic nanoparticles generate heat under an alternating magnetic field by hysteresis loss and/or relaxational loss^22,23^. Magnetic fluid hyperthermia (MFH) has been developed for cancer therapy. It is a largely experimental modality for hyperthermia, and some groups including ours have begun clinical trials for prostate cancer^24^ and melanoma^25^. Generally, nanoparticles of magnetite (Fe_3_O_4_) with a size of 10 nm and magnetic field applicators that generate an alternating magnetic field with an order of tens of kA/m at several 100 kHz have been used for MFH^21,25^. Recently, Manuchehrabadi et al*.* applied magnetic heating technology to cryopreservation of porcine arterial and heat valve tissues, and developed “nano-warming” that employs 10-nm magnetic nanoparticles coated with mesoporous silica and an magnetic field alternating at 100–400 kHz^26^. Nanoparticles can be well dispersed in cryoprotectant solutions, and inductive heating of magnetite nanoparticles enables both uniform and rapid rewarming of vitrified samples independently of the volume. In the present study, we applied these technologies to pluripotent cell production and describe nano-warming of hiPSCs as a scalable cryopreservation technology for regenerative medicine. To the best of our knowledge, this is the first report that has achieved successful cryopreservation of 20-mL hiPSC samples by vitrification, which corresponds to a 100 times larger volume than the gold standard (0.2 mL). Furthermore, we demonstrate that nano-warming enables cryopreservation of hiPSC aggregates prepared by a bioreactor-based approach, which may be beneficial for large-scale production of iPSCs.

## Results

### Limitations of convective warming in scale-up

**Figure 1:**
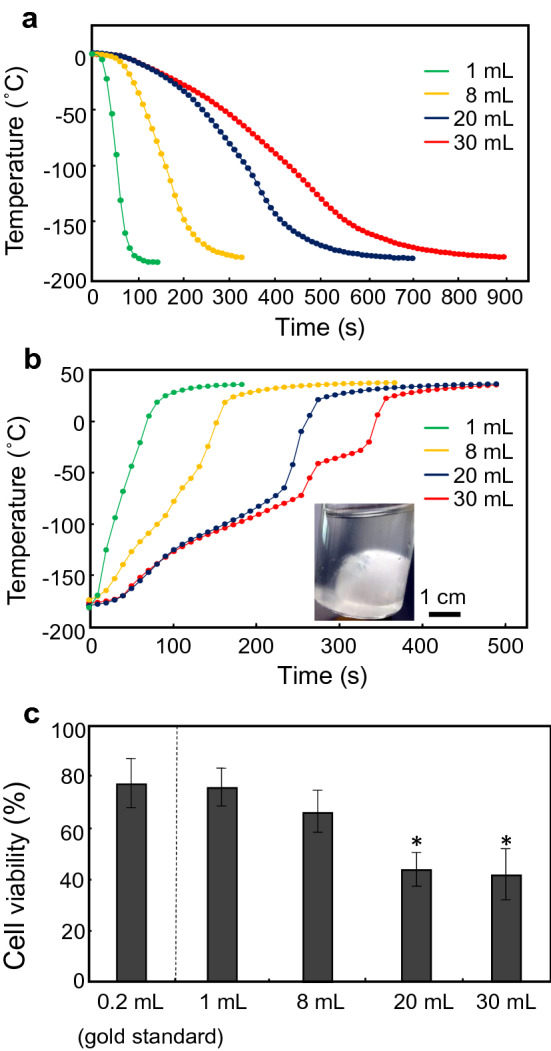
Convective cooling and warming of large volumes of StemCell Keep. Convective cooling for freezing (**a**) and convective warming for thawing (**b**) were carried out by immersing the glass vials in liquid nitrogen and a 37 °C water bath, respectively. Representative data of temperatures at the centre of vials are shown. Green, 1-mL system; yellow, 8-mL system; blue, 20-mL system; red, 30-mL system. A representative photo of ice ball formation induced by devitrification of StemCell Keep during convective warming is also shown in (**b**). (**c**) Effects of convective cooling and warming on the viability of hiPSCs. After freezing and thawing, cell viability was assayed using a cell viability imaging kit based on Hoechst 33342 for live cells and SYTOX green nucleic acid stain for dead cells. Data are expressed as the mean ± SD of three independent experiments. *P < 0.05 compared with the 0.2-mL system (gold standard).

In the cooling process, temperature–time plots of 1- to 30-mL cryoprotectant solutions (StemCell keep) showed smooth curved lines (Fig. 1a), and all samples were vitrified at approximately − 120 °C (glass transition temperature of StemCell Keep^14^) within 900 s. Table 1 shows the cooling rates at the centre of vials calculated by the slopes between 0 and − 100 °C. All samples in a vial with a radius of ≤ 1.6 cm (30-mL vial) achieved a CCR of ≥ 4.9 °C/min for StemCell Keep^15^. However, convective warming of > 20-mL samples failed to rewarm and showed ice crystallization during warming (Fig. 1b). In a time course of warming a 30-mL sample, typical temperature behaviours were observed at 250–270 s (− 74.7 to − 43.7 °C) and 270–320 s (− 43.7 to − 30.9 °C), indicating the recrystallization temperature (− 67.6 °C) and melting temperature (− 35.6 °C) of StemCell Keep^15^, respectively. Furthermore, cell viability was decreased to approximately 40% for both 20- and 30-mL samples (Fig. 1c), whereas a slight decrease of cell viability, but no apparent devitrification, was observed in the 8-mL sample. These results indicate that the CWR of StemCell Keep is around 48.1 °C/min (Table 1), which is consistent with previous reports (10 °C/min < CWR < 50 °C/min)^15,16^.

**Table 1:** Convective cooling and warming rates. *Failure due to crystallization. Data are the mean and SD of three independent experiments.

Volume (radius)	Cooling rate (°C/min)	Warming rate (°C/min)
1 mL (r = 0.5 cm)	127.7 ± 9.0	159.8 ± 10.7
8 mL (r = 1.0 cm)	41.5 ± 1.0	48.1 ± 2.0
20 mL (r = 1.4 cm)	20.5 ± 1.3	*31.2 ± 2.7
30 mL (r = 1.6 cm)	15.0 ± 0.2	*28.1 ± 4.4

### Characterization of nano-warming

**Figure 2:**
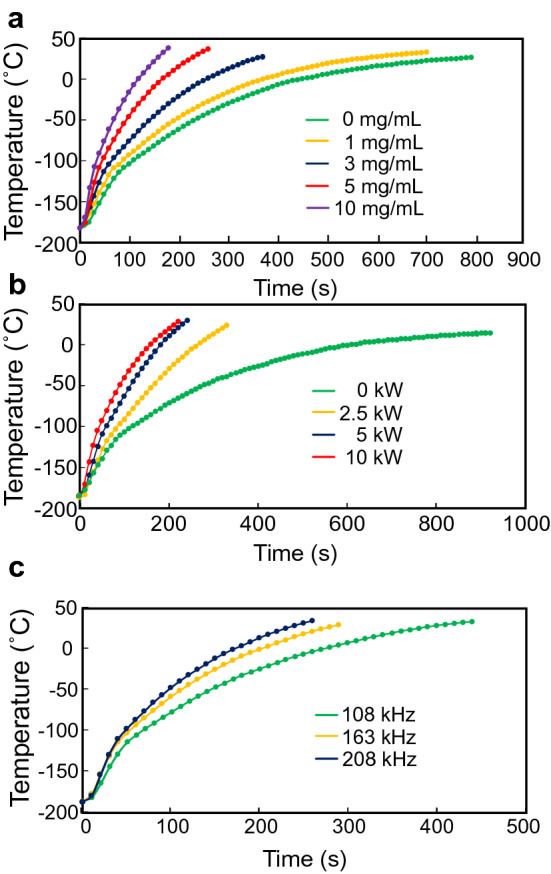
Temperature profiles during alternating magnetic field irradiation. Nano-warming of vitrified StemCell Keep was carried out by inductive heating of magnetite nanoparticles. Representative data of temperatures at the centre of vials are shown. (**a**) Nano-warming power output, 10 kW; magnetite concentration, 0 mg/mL (green), 1 mg/mL (yellow), 3 mg/mL (blue), 5 mg/mL (red), and 10 mg/mL (purple); frequency, 208 kHz. (**b**) Nano-warming power output, 0 kW (green), 2.5 kW (yellow), 5 kW (blue), and 10 kW (red); magnetite concentration, 5 mg/mL; frequency, 208 kHz. (**c**) Nano-warming power output, 10 kW; magnetite concentration, 5 mg/mL; frequency, 108 kHz (green), 163 kHz (yellow), and 208 kHz (blue).

Vitrified 1-mL samples, in which magnetic nanoparticles were dispersed uniformly, were exposed to an alternating magnetic field. We then investigated the effects of the magnetic nanoparticle concentration (Fig. 2a), power output (Fig. 2b), and frequency (Fig. 2c) on nano-warming rates. As a result, the warming rates were increased depending on these three factors, and the CWR of ≥ 48.1 °C/min was achievable at 5 mg/mL magnetite, 10 kW, and 208 kHz (warming rate 65.0 ± 0.2 °C/min) (Table 2). We thus used this nano-warming condition in the following experiments.

**Table 2:** Characterization of nano-warming in a 1-mL system. Data are the mean and SD of three independent experiments.

Magnetite conc (mg/mL)	Power output (kW)	Frequency (kHz)	Warming rate (°C/min)
0	10	208	24.2 ± 2.2
1			29.0 ± 0.6
3			42.3 ± 0.3
5			65.0 ± 0.2
10			95.2 ± 3.1
5	0	208	15.6 ± 0.8
	2.5		40.1 ± 0.4
	5		57.6 ± 0.4
	10		65.0 ± 0.2
5	10	108	41.8 ± 0.7
		163	55.3 ± 0.3
		208	65.0 ± 0.2

**Figure 3:**
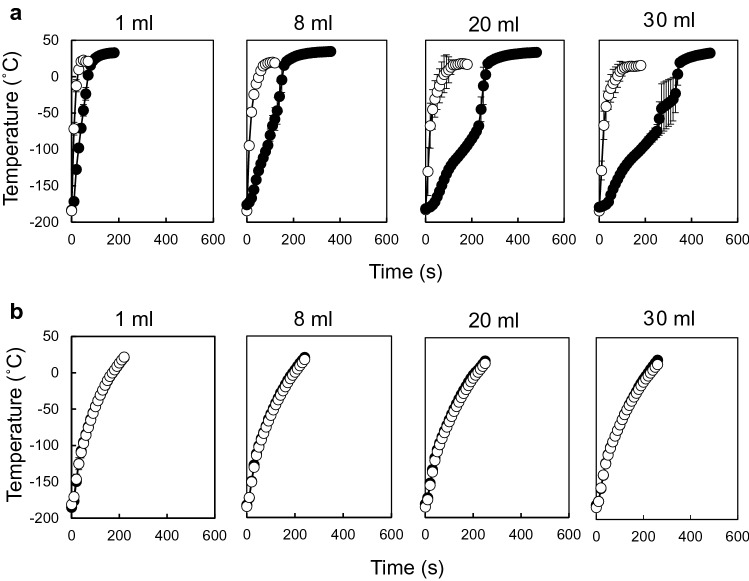
Comparison of convective warming and nano-warming. Convective warming (**a**) and nano-warming (**b**) of vitrified StemCell Keep were carried out. Temperatures at the centre (closed circles) and edge (open circles) of the vials were measured by optical fibre probes. Data are expressed as the mean ± SD (n = 3).

Scale-up of the nano-warming is shown in Fig. 3. Temperature profiles of 1- to 30-mL samples probed at the centre and edge revealed temperature gaps in the convective warming, whereas no temperature gradient between the centre and edge of samples was observed by employing nano-warming. Moreover, the nano-warming rates were independent of the sample volume. These results indicate that nano-warming is scalable and enables both uniform and rapid rewarming.

### Nano-warming of single hiPSCs

**Figure 4:**
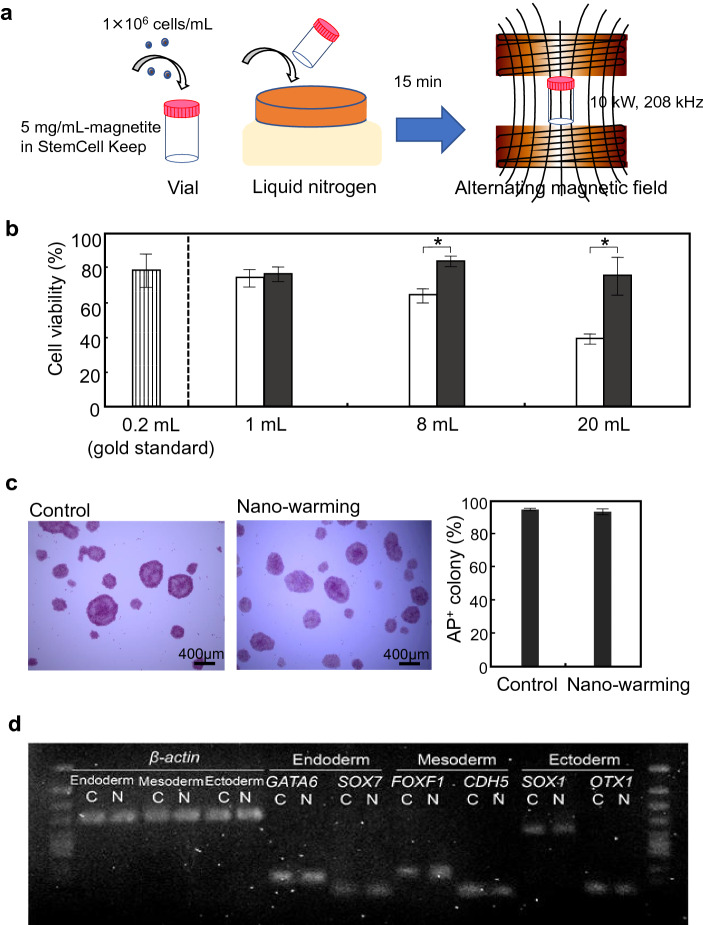
Effects of nano-warming on hiPSC cryopreservation. (**a**) Schematic illustration for cryopreservation of iPSC cells. hiPSCs were applied to a vial at 1 × 10^6^ cells/mL in StemCell Keep containing magnetite nanoparticles at 5 mg/mL. The vial was directly immersed in liquid nitrogen for 15 min. The vial was then irradiated with an alternating magnetic field at 10 kW, 208 kHz. (**b**) Effects of nano-warming on hiPSC viability. After freezing and thawing, cell viability was assayed using a cell viability imaging kit based on Hoechst 33342 for live cells and SYTOX green nucleic acid stain for dead cells. Open columns, convective warming (water bath at 37 °C) of magnetite-loaded StemCell Keep; Closed columns, nano-warming of magnetite-loaded StemCell Keep. Data are expressed as the mean ± SD (n = 3). *P < 0.05. (**c**) Alkaline phosphatase (AP) staining of hiPSC colonies after nano-warming. Control, non-frozen hiPSCs. Data are expressed as the mean ± SD (n = 3). (**d**) RT-PCR analyses of lineage-specific genes. After nano-warming, hiPSCs were cultured under each differentiation condition for the three germ layers: endoderm, mesoderm, and ectoderm. *C* non-frozen control, *N* nano-warming. Relative expression levels normalized to β-actin gene expression are shown below the bands.

Schematic illustration for nano-warming of iPSC cells is shown in Fig. 4a. Figure 4b shows the effect of nano-warming on cell viability. As a control, magnetite nanoparticles (5 mg/mL) were also loaded in the samples (1- to 20-mL) for convective warming. Convective warming of vitrified single hiPSCs affected cell viability. The cell viability of 20-mL samples was 38.5 ± 2.9%, which was similar to the result without magnetite loading (43.2 ± 6.8%; Fig. 1c). hiPSCs after nano-warming were more viable than control cells in larger volume samples (8- and 20-mL). The cell viabilities were 83.0 ± 3.1% and 74.8 ± 10.7%, respectively, which were comparable to that of the gold standard (77.5 ± 9.6%). Moreover, cells after nano-warming (20-mL samples) formed colonies under an undifferentiated culture condition and continued to exhibit alkaline phosphatase (AP) activity (Fig. 4c). Additionally, RT-PCR (Fig. 4d) and qRT-PCR (Supplementary Fig. S1) analyses revealed that cells cultured under differentiation conditions expressed lineage-specific genes such as GATA6 and SOX7 for endoderm, FOXF1 and CDH5 for mesoderm, or SOX1 and OTX1 for ectoderm. These results indicated that the hiPSCs maintained an undifferentiated state and differentiation capability after nano-warming.

### Nano-warming of iPSC aggregate

**Figure 5:**
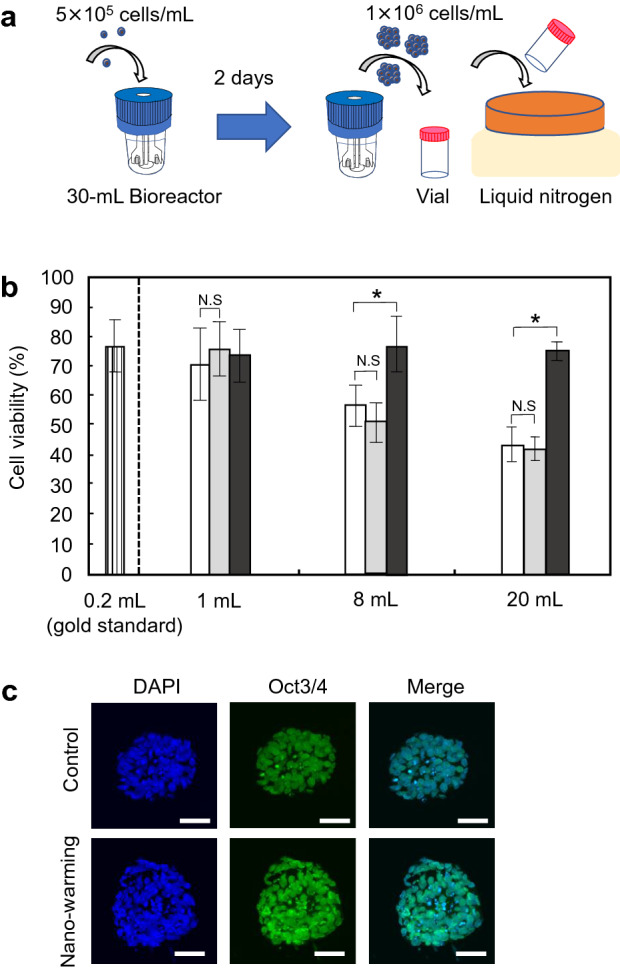
Effects of nano-warming on cryopreservation of hiPSC aggregates. (**a**) Schematic illustration for cryopreservation of iPSC aggregates. hiPSCs were applied to a 30-mL stirred bioreactor at 5 × 10^5^ cells/mL. The cells were cultured for 2 days to form hiPSC aggregates. For cryopreservation of iPSC aggregates, StemCell Keep containing magnetite nanoparticles (5 mg/mL) was added to the cell aggregates at 1 × 10^6^ cells/mL in glass vials, and the vials were directly immersed in liquid nitrogen. (**b**) Effects of nano-warming on the viability of hiPSC aggregates. After freezing and thawing, cell viability was assayed using a cell viability imaging kit based on Hoechst 33342 for live cells and SYTOX green nucleic acid stain for dead cells. White columns, convective warming (water bath at 37 °C) of StemCell Keep; grey columns, convective warming (water bath at 37 °C) of magnetite-loaded StemCell keep; black columns, nano-warming of magnetite-loaded StemCell keep. Data are expressed as the mean ± SD (n = 3). *P < 0.05. *N.S* not significant. (**c**) Florescence images of hiPSC aggregates in non-frozen control (top) and nano-warming (bottom) groups, showing nuclei (DAPI) and Oct3/4. Scale bars, 50 µm.

A bioreactor-based approach for suspension culture of hiPSCs (Fig. 5a) has become a reliable method to obtain a large number of cells. Therefore, we investigated whether cryopreservation of iPSC aggregates could be achieved by the nano-warming technology. Before evaluating nano-warming systems, we compared the effects of vitrification and slow freezing on iPSC aggregates in a 0.2-mL system (Supplementary Fig. S2). The vitrified-and-thawed iPSC aggregates showed a normal morphology. However, cellular boundaries within aggregates that underwent slow freezing were clearly observed under a phase-contrast microscope (Supplementary Fig. S2a). The aggregates subjected to slow freezing were breakable and a relatively small amount of cell adhesion molecule E-cadherin was observed by immunocytochemical analysis (Supplementary Fig. S2b). Furthermore, they showed low viability compared with the vitrified-and-thawed iPSC aggregates (Supplementary Fig. S2c).

Interestingly, convective warming of vitrified iPSC aggregates affected the viability of cells at similar levels to that of single cells (Fig. 5b). The cell viabilities of 20-mL samples with (43.3 ± 5.8%) and without (42.0 ± 4.0%) magnetite nanoparticles were comparable to that of single cells (38.5 ± 2.9%). In addition to single cell cryopreservation, iPSC aggregates after nano-warming were more viable than control cells in larger volume samples. The cell viabilities in 8- and 20-mL systems were 76.9 ± 9.2% and 75.1 ± 3.3%, respectively, which were comparable to that of the gold standard (77.5 ± 9.6%) (Fig. 5b). Immunocytochemical analysis revealed that iPSC aggregates after nano-warming (20-mL samples) expressed pluripotency marker Oct3/4 (Fig. 5c). These results indicated successful cryopreservation of hiPSC aggregates by nano-warming.

## Discussion

Cryopreservation can be used for long-term storage of cells, tissues and, ultimately, organs, and vitrification is considered to be particularly beneficial for such long-term storage. During vitrification, rapid freezing allows the liquid solution to transform to a glassy (amorphous) state, thereby preventing ice crystal formation, which is robust and stable under the glass transition temperature. In the present study, convective cooling for freezing was carried out by immersing the samples in liquid nitrogen for 15 min, and the samples were then applied to rewarming experiments. We conducted a trial in which vitrified iPSCs were prepared at Kyoto University and transported them from Kyoto to Nagoya (> 100 km) using a dry shipping container (cat# DR-2DS, Cryo One, Osaka, Japan). We obtained comparable cell viability (74.5% in a 20-mL system) after nano-warming at Nagoya University. Although vitrified iPSCs were preserved in liquid nitrogen for 2 days in this trial, vitrified samples can be stored indefinitely in theory by employing appropriate containers and facilities. In the future, hiPSC storage and transport will become indispensable technologies for regenerative medicine, and further development is required for practical use.

Slow freezing is based on the mechanism through which cells are hydrated by freezing the cell membrane, which may shrink cells and lead to damage and disruption of cell–cell interactions^27^. In this study, iPSC aggregates that underwent slow freezing showed a clear cell–cell boundary, poor E-cadherin expression, and low cell viability (Supplementary Fig. S2). These results suggest that slow freezing is unsuitable for cryopreservation of multicellular biomaterials such as cell aggregates, tissues, and organs. In the present study, we succeeded in cryopreservation of iPSC aggregates by vitrification, which is considered an important finding for the large-scale production of iPSCs using stirred bioreactors. Here, we focused on scale-up of cryopreservation volumes for iPSC production, whereas cryopreservation of tissues and organs will also require large-scale vitrification. Manuchehrabadi et al*.* demonstrated that nano-warming is effective for tissue cryopreservation in which porcine arteries with a wall thickness of 1–2 mm were evaluated^26^. To achieve successful cryopreservation of more complex and thicker tissues and organs, continuous breakthroughs including perfusion of cryoprotectant solutions in tissues and organs will be necessary.

Toxicology of magnetite nanoparticles is an important issue, and the main requirements for cryopreservation are maintaining cell viability and preserving normal cell behaviours. In the present study, for both single (Figs. 1c, 4b) and aggregated (Fig. 5b) iPSCs, cell viabilities in magnetite-loaded StemCell Keep were comparable to that in StemCell Keep without magnetite nanoparticles. Moreover, iPSCs after nano-warming showed expression of pluripotency markers (Figs. 4c, 5c), and trilineage differentiation was induced successfully (Fig. 4d and Supplementary Fig. S1). Ota et al. reported that the teratoma assay showed that StemCell Keep-cryopreserved human ESCs differentiated into three germ layers^14^. The effects of magnetite nanoparticles on in vivo differentiation of iPSCs remain to be fully elucidated. In our preliminary study of biodistribution, magnetite nanoparticles (90 mg, i.p.) were injected into mice, and none of the 10 mice died during the study. Transient accumulation of magnetite was observed in the liver of the mice, but the magnetite nanoparticles had been cleared from circulation at 10 days after administration^28^. In addition to their high biocompatibility, magnetite nanoparticles can be easily separated by a magnet. In the present study, magnetite nanoparticles were separated with a magnet, and iPSCs after nano-warming were cultured for AP staining (Fig. 4b) and trilineage differentiation (Fig. 4c). We found no detectable magnetite nanoparticles (< 0.02 mg Fe per mL by the potassium thiocyanate method^29^) in iPSCs. Toxicity of magnetic nanoparticles used in nano-warming should be investigated in more detail before entering clinical trials.

In the present study, we demonstrated the success of nano-warming and convective failure for hiPSCs vitrified in a 20-mL system, which corresponded to a 100 times larger volume than the gold standard (0.2 mL). Here, the volume of the cryoprotectant solution was scaled up at the concentration of 1 × 10^6^ cells/mL, and we succeeded in cryopreservation of 2 × 10^7^ cells per vial in a batch. Studies suggest that 1 × 10^9^ functional cells per patient are required for regenerative medicine using hepatocytes, pancreatic β-cells, or cardiomyocytes^5^, and a 1-L cryopreservation system will be required in the future. In the present study, uniform and rapid rewarming rates beyond the CWR were achieved in 1- to 30-mL systems (Fig. 3b), suggesting that nano-warming is a scalable technology. For further scale-up of nano-warming, larger coils of an alternating magnetic field applicator will be required. We previously developed a coil system of 30 cm in diameter for MFH. In the 30-cm coil system, non-specific heating in agarose gel phantoms occurs by eddy current loss at 360 kHz, but we succeeded in reducing non-specific heat generation by lowering the frequency of the alternating magnetic field irradiation to 100–200 kHz (unpublished results). However, achieving the CCR may be more challenging for large-scale vitrification. Rough estimation suggests that StemCell Keep in a vial with a radius of ≤ 2 cm can achieve a CCR of ≥ 4.9 °C/min. Therefore, to vitrify 1 L of solution, the height of the container must be ≥ 80 cm. In the present study, we used glass vials, but their thermal conductivity is known to be low. Most metals cannot be used for nano-warming because they generate heat under an alternating magnetic field. A potential approach includes the use of a container with relatively high thermal conductivity, such as aluminium oxide.

In the current work, we focussed on hiPSC cryopreservation by nano-worming. To confirm the efficacy of this novel method, we conducted nano-warming using another hiPSC line, 253G1^30^ (Supplementary Fig. S3): For 20-mL samples, the cell viability after convective warming was 34.5 ± 3.6%, whereas the cell viability after nano-warming was 67.7 ± 7.8%, which was comparable to that of the gold standard (70.6 ± 3.5%). Also, we believe that nano-worming is applicable to other stem cells, tissue cells and tissue-engineered cell constructs. Recently, Liu et al. used adipose-derived stem cells and showed that nano-warming of cell–alginate hydrogel constructs improved cell survival after cryopreservation^31^. In our preliminary study, cell viability of pancreatic beta cell line MIN6 was improved by nano-warming. Moreover, cell viability of primary mouse islets was improved by nano-warming. In addition to the cell viability, glucose-stimulated insulin secretion was observed at the similar level to the non-frozen control after nano-warming. These results suggest that nano-warming is a potent approach for cryopreservation.

In conclusion, we propose novel methodology for iPSC cryopreservation. Nano-warming is a scalable technology that may facilitate the industrialization of iPSC production and its clinical translation for regenerative medicine.

## Methods

### Preparation of the magnetite-loaded cryopreservation solution

StemCell Keep is a DMSO-free cryopreservation solution containing 6.5 M ethylene glycol, 10% carboxylated poly-l-lysine, and 0.5 M sucrose (BioVerde, Kyoto, Japan)^14^. The magnetite nanoparticles (Fe_3_O_4_; average particle size: 10 nm) were obtained from Dai-ichi High Frequency (Tokyo, Japan). The magnetic characteristics at 796 kA/m (room temperature) were as follows: 2.0 kA/m coercivity, 63.9 Am^2^/kg saturation flux density, and 2.6 Am^2^/kg remanent flux density. To prepare magnetite-loaded cryoprotectant solutions, the magnetite nanoparticles were added to StemCell Keep in cylindrical glass vials (Supplementary Table S1) at the indicated concentrations. The vials were vortexed for 15 min before use.

### Cell culture and preparation for cryopreservation

hiPSCs (clone 201B7^1^) were provided by the RIKEN BRC (Ibaraki, Japan). The cells were cultured on a surface coated with recombinant laminin-511 E8 fragments (iMatrix-511; Nippi, Tokyo, Japan) in culture medium to maintain the undifferentiated state (StemFit AK02N; Ajinomoto, Tokyo, Japan). Cell culture was carried out at 37 °C with 5% CO_2_ in a humidified atmosphere. To cryopreserve single hiPSCs, hiPSC colonies were dissociated into single cells by treatment with Accutase (Innovative Cell Technologies, San Diego, CA) containing 10 μM ROCK inhibitor (Y-27632; Nacalai Tesque, Kyoto, Japan). Cells were counted with a cell counter (Countess II FL; Thermo Fischer Scientific, Waltham, MA) by the trypan blue exclusion method, and added to the cryoprotectant solution at 1 × 10^6^ cells/mL in the glass vials.

For bioreactor experiments, 6 × 10^6^ cells were suspended in medium containing 10 μM ROCK inhibitor and applied to a 30-mL stirred bioreactor (Cat# BMV-S03A; Able, Tokyo, Japan). The agitation speed was 55 rpm. Cells were cultured at 37 °C with 5% CO_2_ in a humidified atmosphere. To cryopreserve hiPSC aggregates, hiPSC aggregates at day 2 of culture were collected from the bioreactor. To determine the number of cells in the aggregates, triplicate 1-mL samples of the cell culture were collected and dissociated into single cells using Accumax (Innovative Cell Technologies). The cells were then counted with the Countess II FL by the trypan blue exclusion method. Cell aggregates corresponding to 1 × 10^6^ cells/mL were added to the cryoprotectant solution in the glass vials.

### Convective cooling and warming

Convective cooling for freezing and convective warming for thawing were carried out by immersing the glass vials or 2-mL polypropylene cryovials (solution volume: 0.2 mL; Corning, New York, NY) as a control (gold standard) in liquid nitrogen and a 37 °C water bath, respectively. Temperatures were measured using optical fibre probes (FX-9020; Anritsu Meter, Tokyo, Japan).

### Nano-warming

An alternating magnetic field was created using a vertical coil (inner diameter 7 cm; length 8 cm) with a transistor inverter (HI-HEATER6020; Dai-ichi High Frequency) operating at 108, 163, or 208 kHz. The maximum levels of power and magnetic field intensity were 10 kW and 31.9 kA/m, respectively. The vial was placed inside the coil, so that the vial was positioned at the centre of the coil. Temperatures during application of the alternating magnetic field were measured by the optical fibre probes. After nano-warming, cells were washed with the culture medium and remaining magnetite nanoparticles were separated with a magnet (diameter 5 cm; height 1 cm; magnetic induction 370 mT).

### Cell viability

Convective cooling for freezing was carried out by immersing the samples in liquid nitrogen for 15 min, and the samples were then warming for thawing. For cell thawing, a water bath for convective warming or alternating magnetic field applicator for nano-warming were turned off when the temperature reached 0 °C to prevent any toxicity from StemCell Keep. After thawing, cells in a 1 mL suspension were collected and their viability was assayed using a ReadyProbes Cell Viability Imaging Kit (Blue/Green) based on Hoechst 33342 for live cells and SYTOX green nucleic acid stain for dead cells (Thermo Fischer Scientific). Cell viability was determined as the percentage of live cells among the total cells counted in five fluorescence images of each sample under a fluorescence microscope (BZ-X810; Keyence, Tokyo, Japan).

### Alkaline phosphate (AP) staining

Cells were seeded in a 6-well culture plate coated with recombinant laminin-511 E8 fragments at 1.3 × 10^4^ cells/well and cultured for 5 days to induce colony formation. After washing with phosphate buffered saline (PBS), the colonies were fixed with 4% paraformaldehyde in PBS for 3 min at room temperature and exposed to a solution containing naphthol AS-MX phosphate (Nacalai Tesque) as a substrate and Fast red TR salt hemi (zinc chloride) salt (Santa Cruz Biotechnology, Dallas, TX) as a coupler for 30 min at 37 °C. AP-positive colonies were determined as the percentage of positive colonies to total colonies counted in five images of each sample under the BZ-9000 microscope.

### Induction of differentiation

After nano-warming, cells were washed with the culture medium and remaining magnetite nanoparticles were separated with the magnet. hiPSCs were induced to differentiate into the three germ layers, ectoderm, mesoderm, and endoderm, using a STEMdiff Trilineage Differentiation Kit (STEMCELL Technologies, Vancouver, Canada), according to the manufacturer’s instructions.

### RT-PCR

For semiquantitative RT-PCR, total RNA was extracted from 1 × 10^6^ cells using an RNAiso Plus Kit (Takara Bio, Shiga, Japan) and reverse transcribed using reverse transcriptase (ReverTra Ace; Toyobo, Osaka, Japan). PCR was performed using the primers for lineage-specific genes listed in Supplementary Table S2. PCR was initiated using a DNA polymerase (G-Taq; Cosmo Genetech, Seoul, Korea) at 94 °C for 2 min, followed by 35 cycles of amplification at 98 °C for 10 s, 57 °C for 30 s, and 68 °C for 30 s, and final extension at 30 °C for 5 min. The PCR products were subjected to electrophoresis on a 2% agarose gel. DNA fragments were visualized by ethidium bromide staining.

### Immunostaining of Oct3/4

hiPSC aggregates were embedded in optical cutting temperature compound (Tissue-Tek, Tokyo, Japan), and thin sections (20 μm thick) were fixed with 4% paraformaldehyde for 10 min at room temperature. The specimens were permeabilized with PBS containing 0.5% Triton X-100 (FUJIFILM Wako Pure Laboratory Chemicals, Osaka, Japan) for 5 min and blocked in Block Ace (Dainippon Sumitomo Pharma, Osaka, Japan) at 4 °C overnight. The specimens were then probed with primary antibodies against Oct3/4 (cat# sc-9081, Santa Cruz Biotechnology) at 4 °C overnight. After washing with Tris-buffered saline, the specimens were immersed in PBS containing 10% Block Ace and an Alexa Fluor 488-conjugated secondary antibody (Thermo Fischer Scientific) for 60 min at room temperature. Cell nuclei were stained with 4′,6-diamidino-2-phenylindol (DAPI, Thermo Fischer Scientific) for 20 min. After washing with PBS, the specimens were observed under a confocal laser-scanning microscope (Olympus, Tokyo, Japan).

### Statistical analysis

Statistical comparisons were made using the Mann–Whitney rank sum test. Values of *P* < 0.05 were considered to be significantly different.

## Supplementary information

Supplementary Information.

## Data Availability

The datasets generated during and/or analysed during current study are available in this article and its Supplementary Information files, or from the corresponding author on request.
