# Supplementary material for: Magnetic heating of nanoparticles as a scalable cryopreservation technology for human induced pluripotent stem cells
Source: Sci Rep. 2020 Aug 12;10:13605. doi: 10.1038/s41598-020-70707-6 (PMC7423927; doi:10.1038/s41598-020-70707-6)
Supplement: Supplementary file 1 — Supplementary Information. [file 41598_2020_70707_MOESM1_ESM.doc]

Supplementary Information

Magnetic heating of nanoparticles as a scalable cryopreservation technology for human induced pluripotent stem cells

Akira Ito^1,2^,*, Kantaro Yoshioka^3^, Shinya Masumoto^3^, Keiichiro Sato^3^, Yuki Hatae^3^, Tomoki Nakai^1^, Takashi Yamazaki^1^, Masazumi Takahashi^4^, Shota Tanoue^4^ & Masanobu Horie^5^

^1^Department of Chemical Systems Engineering, School of Engineering, Nagoya University, Furo-cho, Chikusa-ku, Nagoya 464-8603, Japan. ^2^Precursory Research for Embryonic Science and Technology (PRESTO), Japan Science and Technology Agency (JST), 4-1-8 Honcho, Kawacughi, Saitama, 332-0012, Japan. ^3^Department of Chemical Engineering, Faculty of Engineering, Kyushu University, 744 Motooka, Nishi-ku, Fukuoka 819-0395, Japan. ^4^Technical Department, Dai-Ichi High Frequency Co., Ltd. 1-45 Mizue-cho, Kawasaki-ku, Kawasaki 210-0866, Japan. ^5^Division of Biochemical Engineering, Radioisotope Research Center, Kyoto University, Yoshida Konoe-cho, Sakyo-ku, Kyoto 606-8501, Japan.

*Correspondence and requests for materials should be addressed to A. I.

(Email: ito.akira@material.nagoya-u.ac.jp)

**Methods**

**Quantitative RT-PCR.** Total RNA was extracted from 1 × 10^6^ cells using an RNAiso Plus Kit (Takara Bio, Shiga, Japan) and reverse transcribed using reverse transcriptase (ReverTra Ace; Toyobo, Osaka, Japan). Quantitative PCR was performed with SYBR green PCR Master Mix (Thermo Fischer Scientific, Waltham, MA) and analysed with a StepOnePlus Real-Time PCR System (Thermo Fischer Scientific). The sequences of primers for lineage-specific genes are listed in Supplementary Table S3. Gene expression was normalized to that of GAPDH gene as the internal control and quantified by the ΔΔCt method.

**Slow freezing of hiPSC aggregates.** hiPSC aggregates cultured in the 30-mL bioreactor for 2 days were prepared as described in the main text. Triplicate 1 mL samples of the cell culture were collected to determine the number of cells. The cell samples were dissociated into single cells using Accumax (Innovative Cell Technologies, San Diego, CA) containing 10 μM ROCK inhibitor (Nacalai Tesque, Kyoto, Japan) and then counted with a Countess II FL (Thermo Fischer Scientific, Waltham, MA) by the trypan blue exclusion method. Stem Cell Banker (Nihon Zenyaku Kogyo, Fukushima, Japan) was added to the cell aggregates at 1 × 10^6^ cells/mL (total volume: 0.2 mL) in 2-mL polypropylene cryovials (Corning, New York, NY). The cryovials were placed in a freezing container (Mr. Frosty; Thermo Fischer Scientific) and cooled in a freezer at -80 °C overnight. Convective warming for thawing was carried out by immersing the vials in a 37 °C water bath.

**Immunostaining of E-cadherin.** hiPSC aggregates were embedded in optical cutting temperature compound (Tissue-Tek, Tokyo, Japan), and thin sections (20 μm thick) were fixed with 4% paraformaldehyde for 10 min at room temperature. The specimens were permeabilized with PBS containing 0.5% Triton X-100 (FUJIFILM Wako Pure Laboratory Chemicals, Osaka, Japan) for 5 min and blocked in Block Ace (Dainippon Sumitomo Pharma, Osaka, Japan) at 4 °C overnight. The specimens were then probed with a primary antibody against E-cadherin (cat# sc-21791, Santa Cruz Biotechnology, Dallas, TX) at 4 °C overnight. After washing with Tris-buffered saline, the specimens were immersed in PBS containing 10% Block Ace and an Alexa Fluor 488-conjugated secondary antibody (Thermo Fischer Scientific) for 60 min at room temperature. In addition, cell nuclei were stained with DAPI (Thermo Fischer Scientific) for 20 min. After washing with PBS, the specimens were observed under a confocal laser-scanning microscope (FV1000; Olympus, Tokyo, Japan).

**Nano-warming of 253G1 cells.** hiPSCs (clone 235G1) were provided by the RIKEN BRC (Ibaraki, Japan). The cells were cultured on a surface coated with recombinant laminin-511 E8 fragments (iMatrix-511; Nippi, Tokyo, Japan) in culture medium to maintain the undifferentiated state (StemFit AK02N; Ajinomoto, Tokyo, Japan). Cell culture was carried out at 37 °C with 5% CO2 in a humidified atmosphere. To cryopreserve cells, hiPSC colonies were dissociated into single cells by treatment with Accutase (Innovative Cell Technologies, San Diego, CA) containing 10 μM ROCK inhibitor (Y-27632; Nacalai Tesque, Kyoto, Japan). Cells were added to the cryoprotectant solution at 1 × 10^6^ cells/mL in the glass vials. Convective cooling for freezing and convective warming for thawing were carried out by immersing the 20-mL glass vials or 2-mL polypropylene cryovials (solution volume: 0.2 mL; Corning, New York, NY) as a control (gold standard) in liquid nitrogen and a 37 °C water bath, respectively. For nano-warming, an alternating magnetic field was created using a vertical coil (inner diameter: 7 cm; length: 8 cm) with a transistor inverter (HI-HEATER6020; Dai-ichi High Frequency) operating at 208 kHz. The vial was placed inside the coil, so that the vial was positioned at the centre of the coil. After thawing, cells in a 1 mL suspension were collected and their viability was assayed using a ReadyProbes Cell Viability Imaging Kit (Blue/Green) based on Hoechst 33342 for live cells and SYTOX green nucleic acid stain for dead cells (Thermo Fischer Scientific). Cell viability was determined as the percentage of live cells among the total cells counted in five fluorescence images of each sample under a fluorescence microscope (BZ-X810; Keyence, Tokyo, Japan).

Table S1. Glass vials used in this study.

| Name of system | Inner diameter  (mm) | Outer diameter  (mm) | Height  (mm) | Volume of solution  (mL) |
| --- | --- | --- | --- | --- |
| 1-mL | 10.6 | 12 | 35 | 1 |
| 8-mL | 19 | 21 | 45 | 8 |
| 20-mL | 27.6 | 30 | 63 | 20 |
| 30-mL | 32.4 | 35 | 78 | 30 |

Supplementary Table. S1 Ito et al.

Table S2. Primer sequences for RT-PCR analyses.

| Target gene | Primer sequence |
| --- | --- |
| β-actin (511 bp) | FW: 5’-CTC TTC CAG CCT TCC TTC C-3’  RV: 5’-CAC CTT CAC CGT TCC AGT TT-3’ |
| GATA6 (161 bp) | FW: 5’-TGT GCA GCA ATG CTT GTG GAC TC-3’  RV: 5’-AGT TGG AGT CAT GGG AAT GG-3’ |
| SOX7 (110 bp) | FW: 5’-GCC AAG GAC GAG AGG AAA C-3’  RV: 5’-CTC TTC TGG GAC AGC GTC A-3’ |
| FOXF1 (194 bp) | FW: 5’-ACA GCC GCG CCT CTT ATA TC-3’  RV: 5’-CTC CTT TCG GTC ACA CAT GC-3’ |
| CDH5 (114 bp) | FW: 5’-GAT CAA GTC AAG CGT GAG TCG-3’  RV: 5’-AGC CTC TCA ATG GCG AAC AC-3’ |
| SOX1 (423 bp) | FW: 5’-GCG GAA AGC GTT TTC TTT G-3’  RV: 5’-TAA TCT GAC TTC TCC TCC C-3’ |
| OTX1 (121 bp) | FW: 5’-CAC TAA CTG GCG TGT TTC TGC-3’  RV: 5’-AGG CGT GGA GCA AAA TCG-3’ |

Supplementary Table. S2 Ito et al.

Table S3. Primer sequences for qRT-PCR analysis.

| Target gene | Primer sequence |
| --- | --- |
| GAPDH | FW: 5′- CTACCCCCAATGTGTCCGTC -3′  RV: 5′- GCTGTTGAAGTCGCAGGAGAC -3′ |
| GATA6 | FW: 5’- GACTTGCTCTGGTAATAG-3’  RV: 5’- CTGTAGGTTGTGTTGTGG-3’ |
| SOX7 | FW: 5’- TGTAGCCACCCCCTGGGCTC-3’  RV: 5’- TGGGGGACAGCCGGGTACAG-3’ |
| FOXF1 | FW: 5’- CGTATCTGCACCAGAACAGC-3’  RV: 5’- ACTACCACCAGCAGGTCACC-3’ |
| CDH5 | FW: 5’- ACTACCACCAGCAGGTCACC-3’  RV: 5’- TCGACGATGAAGCTGTATTG-3’ |
| SOX1 | FW: 5’- TGTTGGCATCTAGGTCTTGGCTCA-3’  RV: 5’- TGTGCACGAAGCACCTGCAATAAG-3’ |
| OTX1 | FW: 5’- CACTAACTGGCGTGTTTCTGC-3’  RV: 5’- GGCGTGGAGCAAAATCG-3’ |

Supplementary Table. S3 Ito et al.

Supplementary Figure S1 Ito et al.


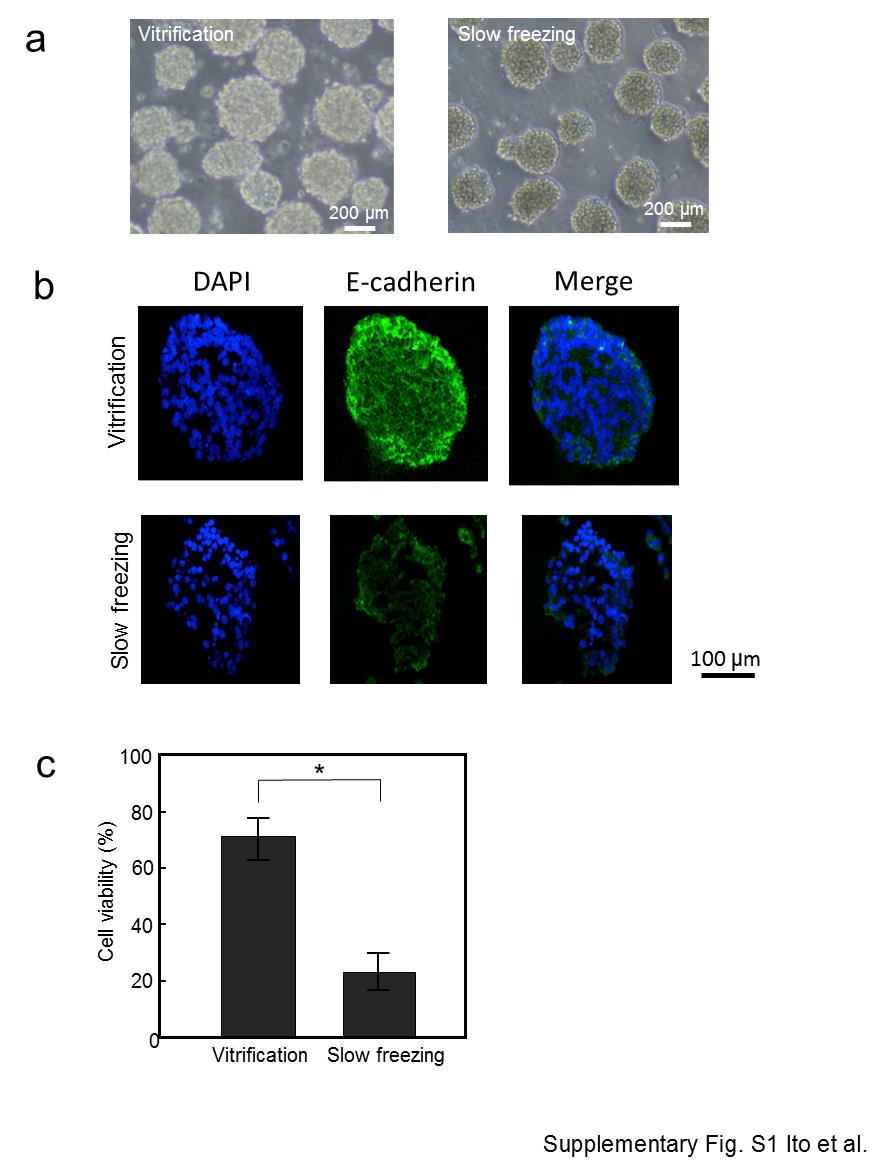


Supplementary Figure S2 Ito et al.

Supplementary Figure S3 Ito et al.

**Figure legends**

**Supplementary Figure S1│qRT-PCR analysis.** After nano-warming, hiPSCs were cultured under each differentiation condition for the three germ layers: endoderm, mesoderm, and ectoderm. qRT-PCR was used to assess trilineage differentiation marker genes [endodermal (GATA6 and Sox7), mesodermal (FoxF1 and CDH5) and ectoderm (Sox1 and OTX1)]. Open columns, non-frozen control; closed columns, nano-warming. Relative expression levels normalized to GAPDH gene expression are shown. Data are expressed as the mean ± SD (n=3).

**Supplementary Figure S2│Comparison of slow freezing and vitrification.** (**a**) Bright field micrographs of hiPSC aggregates after vitrification (**left**) and slow freezing (**right**). (**b**) Florescence images of hiPSC aggregates after vitrification (**top**) and slow freezing (**bottom**), showing nuclei (DAPI) and E-cadherin. (**c**) Viability of hiPSC aggregates after cryopreservation. After freezing and thawing, cell viability was assayed by counting cells with the trypan blue exclusion method using the Countess II FL. Data are expressed as the mean ± SD (n=3). **P* < 0.05.

**Supplementary Figure S3│Effects of nano-warming on 235G1 cell cryopreservation.** 253G1 cells were applied to a vial at 1 × 10^6^ cells/mL in StemCell Keep containing magnetite nanoparticles at 5 mg/mL. The vial was directly immersed in liquid nitrogen for 15 min. The vial was then irradiated with an alternating magnetic field at 10 kW, 208 kHz. After freezing and thawing, cell viability was assayed using a cell viability imaging kit based on Hoechst 33342 for live cells and SYTOX green nucleic acid stain for dead cells. Open column, convective warming (water bath at 37 °C); Closed column, nano-warming. Data are expressed as the mean ± SD (n=3). **P* < 0.05.
